# Supplementary material for: ICAM-1-suPAR-CD11b Axis Is a Novel Therapeutic Target for Metastatic Triple-Negative Breast Cancer
Source: Cancers (Basel). 2023 May 12;15(10):2734. doi: 10.3390/cancers15102734 (PMC10216673; doi:10.3390/cancers15102734)
Supplement: Supplementary file 1 [file cancers-15-02734-s001.zip › cancers-2301662-supplementary.pdf]

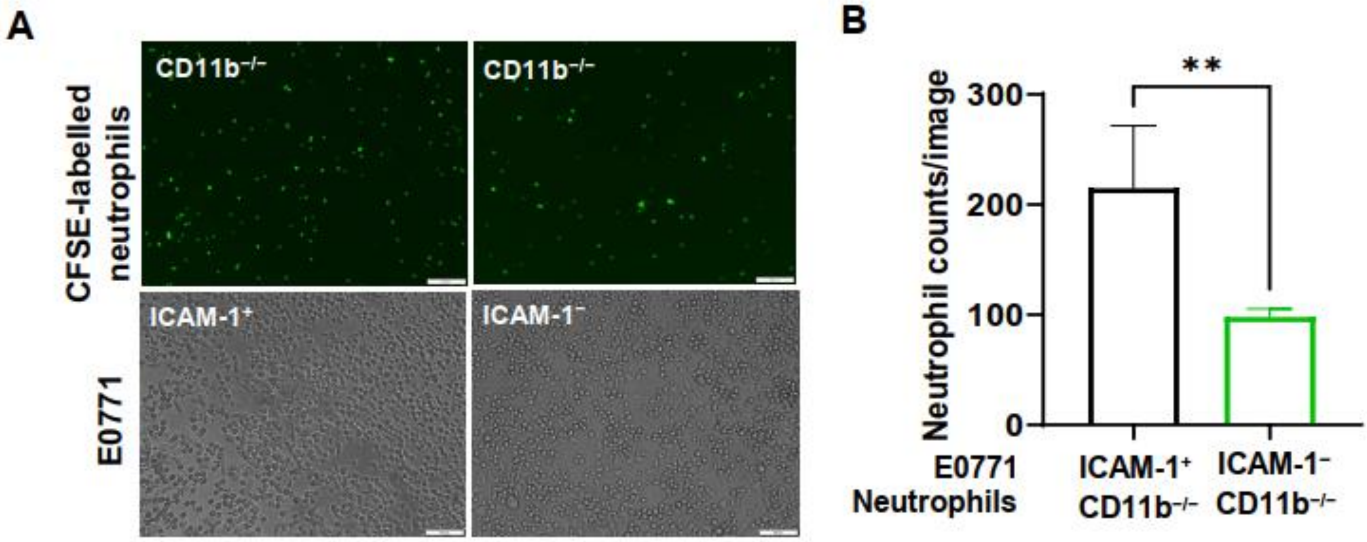

Figure S1. ICAM-1 enhances the binding ability of tumor cells to neutrophils. BM Neutrophils isolated from $CD11b^{-/-}$ mice were labeled with CFSE, and cocultured with ICAM-1^{+} or ICAM-1^{-} E0771 cells for 2h. The binding of neutrophils with tumor cells was imaged **(A)** and quantitated **(B)** (t-test, ** $p<0.01$, n=5).

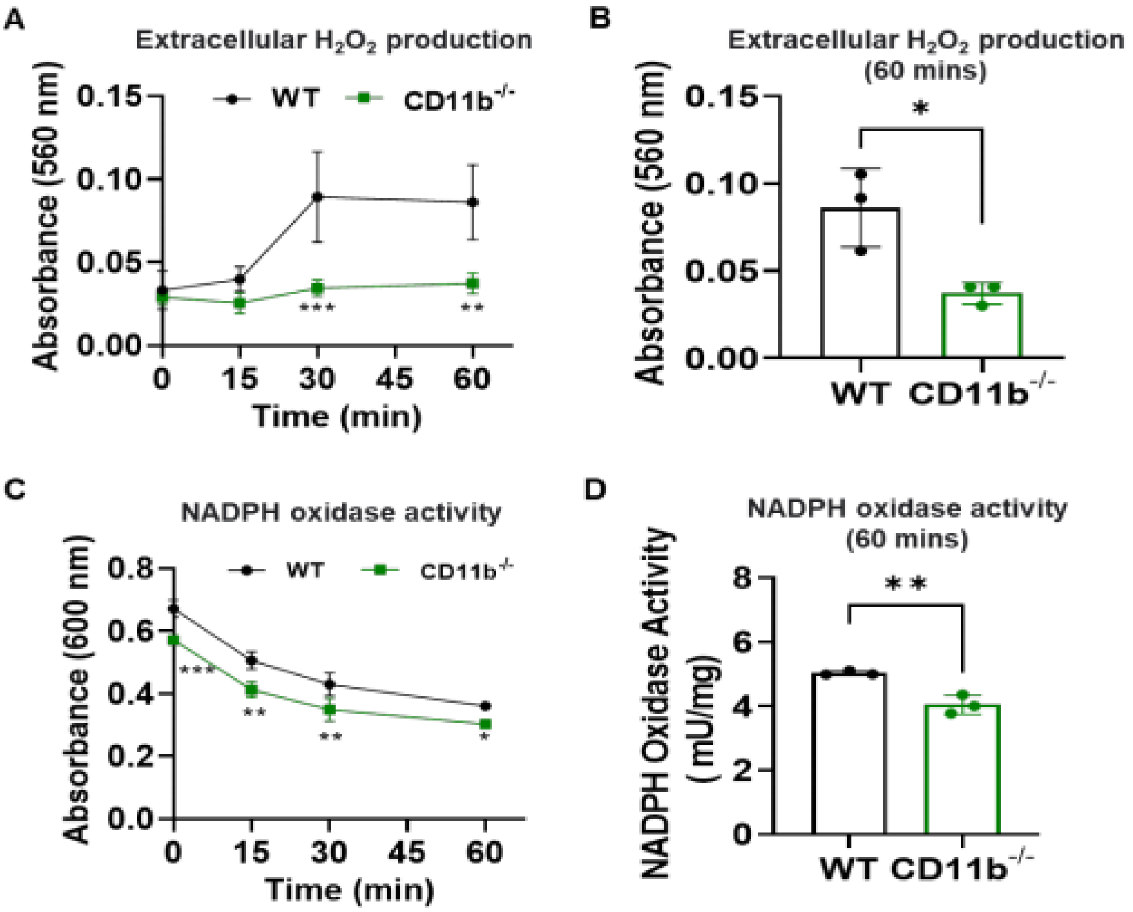

Figure S2. CD11b promotes H_2O_2 production from neutrophils via activation of NADPH oxidases. (A, B) The bone marrow neutrophils were isolated from $CD11b_{-/-}$ and WT C57BL/6 mice. The extracellular H_2O_2 production from neutrophils was measured by Amplex™ Red Hydrogen Peroxide/Peroxidase Assay Kit. The absorbance (OD_{560} nm) was measured in kinetic mode for 60 minutes (mins) using the Promega™ GloMax® Plate Reader **(A)** and the extracellular H_2O_2 production at 60 mins was quantitated **(B)**. t-test, $*p < 0.05$, $**p < 0.01$, $***p < 0.001$ (n=3). **(C, D)** The bone marrow neutrophils were isolated from $CD11b_{-/-}$ and WT C57BL/6 mice, and the NADPH oxidase (NOX) activity was measured by NADPH Oxidase Activity Assay Kit. The absorbance (OD_{600} nm) was measured in kinetic mode for 60 mins **(C)**, and the NADPH oxidase activity (mU/mg) at 60 mins was calculated **(D)**. t-test, $*p < 0.05$, $**p < 0.01$, $***p < 0.001$ (n=3).

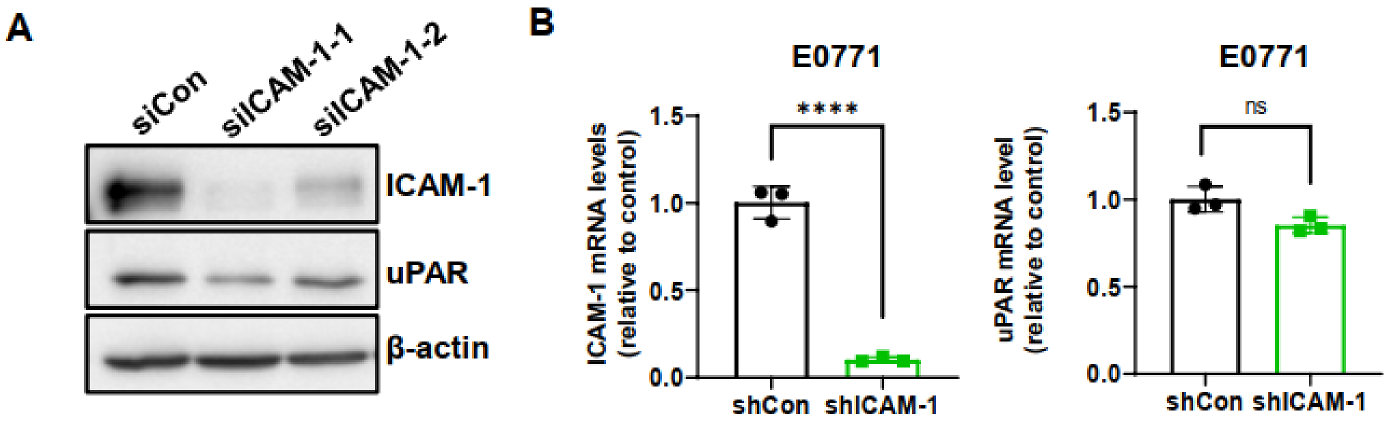

Figure S3. ICAM-1 does not regulate uPAR mRNA levels. (**A)** MDA-MB-231 cell were transfected with two differen siICAM-1 RNAs. The cells were collected after 48 hours, and the expressions of ICAM-1 and uPAR were measured by Western blotting. (**B)** ICAM-1 does not regulate uPAR mRNA levels. The ICAM-1 and uPAR mRNA levels in control (shCon and ICAM-1 knockdown (shICAM-1) E0771 cells were determined by RT-PCR. t-test, ns, not significant; ****p <0.0001 (n=3).

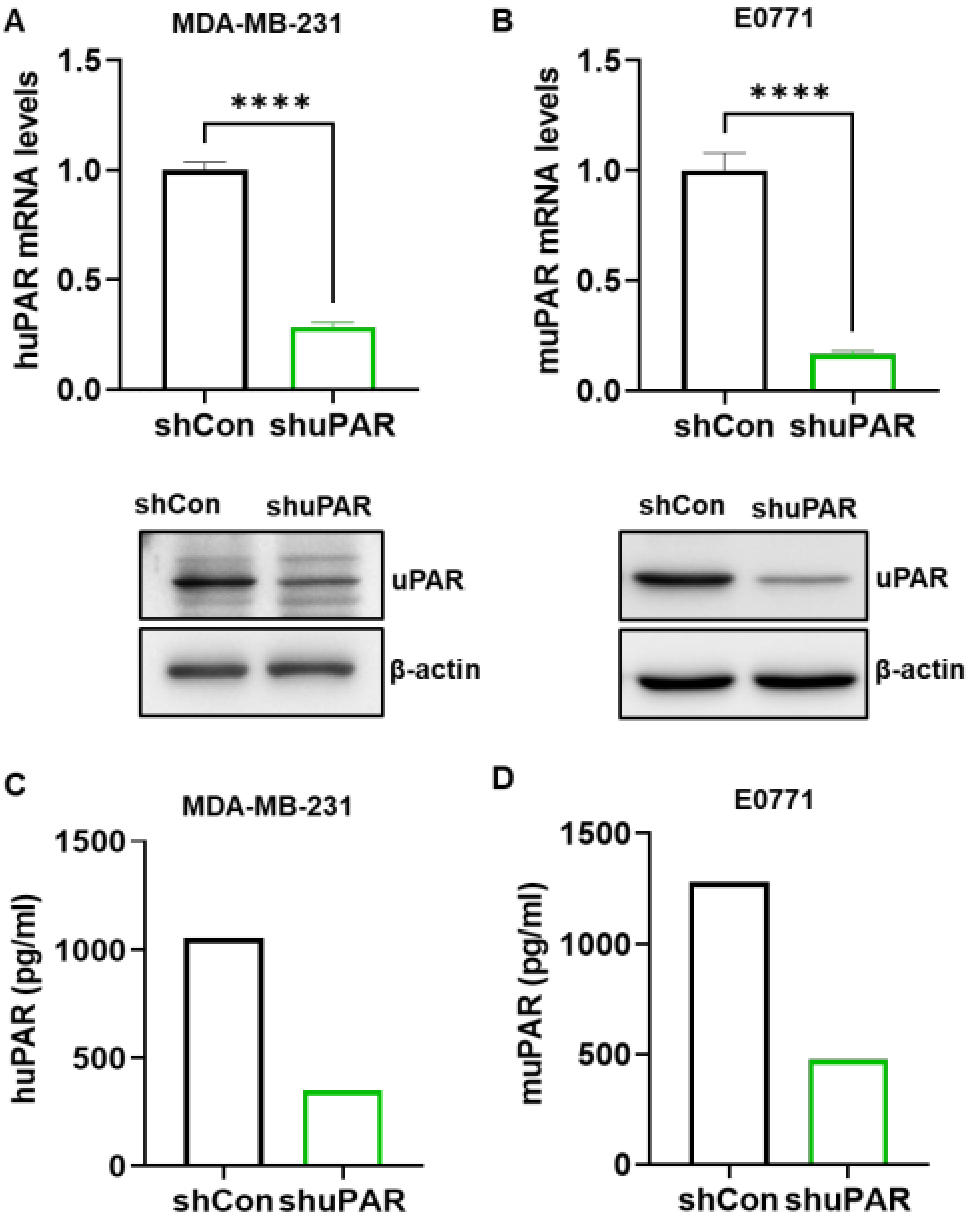

Figure S4. uPAR knockdown efficacy in MDA-MB-231 and E0771 cells. (A&B) The efficiency of uPAR knockdown in MDAMB-231 (A) and E0771 (B) cells. The uPAR mRAN (upper) and protein (lower) levels were determined by RT-PCR and Western blotting, respectively. t-test, ****p <0.0001 (n=3). (C&D) The secreted human uPAR and mouse uPAR in the supernatants from cultured control and uPAR knockdown MDA-MB-231 (left) and E0771 (right) cells were measured by uPAR ELISA kit. Representative data from two independent experiments were shown.

Table S1. PCR primers

Primer	Sequence (5′ - 3′)
hICAM-1F	ATGCCCAGACATCTGTGTCC
hICAM-1R	GGGGTCTCTATGCCCAACAA
huPAR F	TGTAAGACCAACGGGGATTGC
huPAR R	AGCCAGTCCGATAGCTCAGG
hGAPDH F	ACAACTTTGGTATCGTGGAAGG
hGAPDH R	GCCATCACGCCACAGTTTC
mICAM-1F	GTGATGCTCAGGTATCCATCCA
mICAM-1R	CACAGTTCTCAAAGCACAGCG
muPAR F	CAGAGCTTTCCACCGAATGG
muPAR R	GTCCCCGGCAGTTGATGAG
mGAPDH F	AGGTCGGTGTGAACGGATTTG
mGAPDH R	TGTAGACCATGTAGTTGAGGTCA

Table S2. PCR setting to synthesize cDNA for mRNA amplification

Steps	1	2	3	4
Temperature (°C)	25	37	85	4
Time (min)	10	120	5	∞

Table S3. qRT-PCR setting to amplify mRNA

	Hold Stage		PCR Stage			Melt Curve	
**Steps**	1	2	1	2	1	2	3
**Temperature (°C)**	50	95	95	60	95	60	95
**Time**	2 min	10 min	15 sec	30 sec	15 sec	1 min	15 sec
**cycle**	× 1			× 40		× 1	

Table S4. Antibodies

Ab name	Application	Dilution factor	Source	Cat. No.
**PE-anti-ICAM-1**	IF	1:100	BD	555511
**APC-anti-CD45**	IF	N/A	CellSearch	7900001
**PE-anti-cytokeratins**	IF	N/A	CellSearch	7900001
**ICAM-1**	WB	1:1000	Proteintech	10020-1-AP
**uPAR**	WB	1:1000	Proteintech	10286-1-AP
**actin**	WB	1:2000	Thermo Fisher	MA5-11869
**Ly6G**	IHC	1:500	BD	551459
**APC anti-mouse/human CD11b**	Flow	1:500	BioLegend	101212
**APC/Cyanine7 anti-mouse Ly-6G**	Flow	1:500	BioLegend	127624
